# First-Year Students Background and Academic Achievement: The Mediating Role of Student Engagement

**DOI:** 10.3389/fpsyg.2019.02669

**Published:** 2019-12-09

**Authors:** Luísa Ribeiro, Pedro Rosário, José Carlos Núñez, Martha Gaeta, Sonia Fuentes

**Affiliations:** ^1^Faculdade de Educação e Psicologia, Centro de Investigação para o Desenvolvimento Humano, Universidade Católica Portuguesa, Porto, Portugal; ^2^Escola de Psicologia, Universidade do Minho, Braga, Portugal; ^3^Facultad de Psicología, Universidad de Oviedo, Oviedo, Spain; ^4^Facultad de Ciencias Sociales y Humanidades, Universidad Politécnica y Artística de Paraguay, San Ignacio, Paraguay; ^5^Facultad de Educación, Universidad Popular Autónoma del Estado de Puebla, Puebla, Mexico; ^6^Facultad de Educación, Universidad Central de Chile, Santiago, Chile

**Keywords:** cognitive engagement, behavioral engagement, academic achievement, first-year students, structural equation modeling

## Abstract

The current study aimed to analyze the relationships between students’ background variables (students’ academic preparation and sociocultural status), students’ cognitive and behavioral engagement, and an outcome variable (academic achievement). One sample of 380 first-year students who were studying in different scientific areas participated in the study. Students answered a questionnaire at the beginning and at the end of their first semester in college. To increase ecological validity, students’ cognitive and behavioral engagement and academic achievement were assessed using a specific curricular subject of the course as a reference. Students’ grades were collected through academic services. Data from both time points were analyzed with a structural equation model (SEM), and data showed a goodness of fit of SEM in both time points. Findings indicate that cognitive and behavioral engagement mediated the relationship between students’ background variables and their academic achievement. The analysis of both SEM allows us to understand that academic achievement at the end of the semester is closely related to what happens at the beginning of the semester (e.g., approach to learning, study time). Thus, promoting students’ engagement at the beginning of the semester should be considered a priority, as the first part of the first semester represents a critical period for students and for their integration in college. Thus, universities should consider improving their mechanisms of collecting information to allow for early identification, support, and monitoring of students at risk of dropping out, showing high level of disengagement and low academic achievement.

## Introduction

Educational research has been examining the factors that influence and correlate with college students’ academic achievement and psychosocial development (Winne and Nesbit, 2010; Woitschach et al., 2017; Meens et al., 2018; Willems et al., 2019). The recent massification of higher education raises new challenges concerning success in university, once the students’ previous experiences, their sociocultural roots, and academic needs are more diverse. First year in higher education has been identified in the literature as being a critical year for the students’ future success, retention, and persistence at the academy (Merhi et al., 2018; Baik et al., 2019). First-year students not only develop attitudes toward their academic courses that are likely to shape their future engagement in the field, but they also develop perceptions about themselves as students (Hernández-Pina et al., 2006; Lerdpornkulrat et al., 2018; Soytürk and TepeköylüÖztürk, 2019). In addition, dropout occurs more frequently in first year (Razouki et al., 2019), resulting in social and individual consequences (Aina, 2013; Meens et al., 2018). Research on the dynamics of students’ nonengagement during the first semester is important, as it can be considered a risk factor that affects students’ academic success (van der Meer et al., 2018).

Thus, the current study aimed to analyze the relationships between predictor variables (students’ academic preparation and sociocultural status), students’ cognitive and behavioral engagement, and an outcome variable (academic achievement). A structural equation model (SEM) was fit at the beginning and at the end of first semester of first year of university.

### Students’ Background: Academic Preparation and Sociocultural Status

In this section, literature on academic preparation (prior knowledge and language skills) and on sociocultural status (cultural capital and first-generation status) is reviewed.

According to the literature, students’ prior knowledge plays an important role in academic achievement (Trigwell, 2010; Masui et al., 2014; Niessen et al., 2018; Lin and Liou, 2019). Some authors point it out as the major predictor of college students’ behaviors during their first year (Zwick and Sklar, 2005). According to Plant et al. (2005), prior knowledge has an indirect impact on academic achievement at the end of semester due to its influence on the quantity and type of new learning students need to undertake to reach a high level of mastery. Students’ language skills show a positive correlation with students’ outcomes (Savolainen et al., 2008), but the literature points to the lack of basic skills such as text comprehension in many first-year students (Hungwe, 2019). Despite the importance of this academic competence, crucial for coping with the academic challenges that students face at university, the analyses of the role of reading, understanding, and writing skills in students’ academic outcomes have received limited attention.

Research acknowledges the role played by students’ cultural capital in their academic outcomes (van de Werfhorst, 2010; Nichols and Islas, 2016). Cultural capital is particularly relevant for students from less advantaged family backgrounds (Roksa and Robinson, 2017). First-generation students, meaning students whose parents did not attend a higher education institution (Choi and Rhee, 2014; Nichols and Islas, 2016), have been identified as a unique demographic group. These students can be considered at risk because, when compared with their colleagues, they are more likely to display lower levels of engagement at university (Kuh et al., 2007), academic achievement (Engle, 2007), and are more likely to drop out (Aina, 2013). However, James et al. (2010) found that first-generation students, compared with their counterparts, have clearer objectives, are more consistent working throughout the semester, and manage their academic work load more strategically, in spite of feeling overwhelmed by the number of tasks they have to complete.

### Student Engagement

Engagement has been pointed out as a major dimension of students’ level and quality of learning, namely in what concerns the improvement of their academic achievement, their persistence versus dropout, as well as their personal and cognitive development (Pascarella and Terenzini, 2005; Kuh et al., 2008; Skinner et al., 2009; Kahu, 2013; Rosário et al., 2016). Engagement was defined by Astin (1999) as “the amount of physical and psychological energy that the student devotes to the academic experience” (p. 518). However, recent research reinforces the idea that student engagement is a complex, multifaceted, and multidimensional meta-construct (Fredricks et al., 2004; Zepke and Leach, 2010).

Cognitive engagement concerns the investment in learning, the effort implicated in understanding complex ideas and one’s mastering of challenging skills (Fredricks et al., 2004; Núñez et al., 2006; Trowler, 2010). According to Lewis et al. (2011), the cognitive dimension of engagement lacks attention from the literature. Several authors have related cognitive engagement to students’ use of cognitive strategies and considered the adoption of a deeper approach to learning, centered on understanding and connecting ideas, as these are both considered signs of students’ investment (Fredricks et al., 2004; Harris, 2011; Korhonen et al., 2017). When adopting a deep approach, students attribute a personal meaning to the contents, by relating new ideas to their previous knowledge and experiences in the surrounding world. When a surface approach is adopted, students are likely to focus on fulfillment of the requirements of a certain task with minimum effort, for example, using strategies based on memorization to reproduce the learning material later on (Entwistle, 2009; Trigwell, 2010). The adoption of a particular approach to learning, either deep or surface one, represents the students’ answer to personal or contextual factors related to specific subjects and to the perceived demands concerning a certain learning task (Rosário et al., 2013a). This responsive dynamic has been related to the student engagement construct as well, which is considered malleable and situational and is influenced by individual and contextual factors (Fredricks et al., 2004; Kahu, 2013).

Behavioral engagement refers to the observable dimensions of student engagement, namely, the fulfillment of the rules, attendance at classes, and the accomplishment of the tasks assigned by the teachers (Fredricks et al., 2004; Archambault et al., 2009a,b; Trowler, 2010). Analyzing students’ study time constitutes an important dimension of behavioral engagement, as it allows for a better understanding of the extent to which the academic outcomes derive from students’ decisions made after entering higher education or from previous background factors influencing them before their arrival at university. Class attendance is also an important dimension of behavioral engagement (Appleton et al., 2006; Kahu, 2013). To obtain high-quality academic outcomes, students are expected to attend the majority of classes, where contents are taught, and specific instructions about the material to study and skills to practice are provided (Plant et al., 2005).

### Purpose of the Study

Several authors have pointed out the importance of analyzing antecedents and consequences of student engagement, placing this variable as a mediator between predictive factors and outcomes (Fredricks et al., 2004; Steele and Fullagar, 2009; Pike et al., 2012; Kahu, 2013). Structural equation modeling (SEM) fit on the current study intends to bring relevant contributions while incorporating student engagement as a mediating factor in the relationship between background variables (i.e., academic preparation and sociocultural status) and outcome variables (i.e., academic achievement). Besides, in response to limitations in existing literature, engagement in the current study was analyzed in two dimensions (the cognitive dimension, through approaches to learning, and the behavioral dimension; Fredricks et al., 2004). The variables considered in the model are in line with research in this area, hypothesizing the influence of cognitive engagement on behavioral engagement (Archambault et al., 2009a,b; Kahu, 2013). The current study tested the model fit during the first weeks of the semester, as this is the period of time in which is important to identify early indicators of nonengagement (van der Meer et al., 2018). The stability of the model in two different time points (at the beginning and at the end of first semester of first year) has also been tested to examine whether the experience of attending a semester at university changed the relationship between variables.

### Hypothetical Model

**Figure 1:**
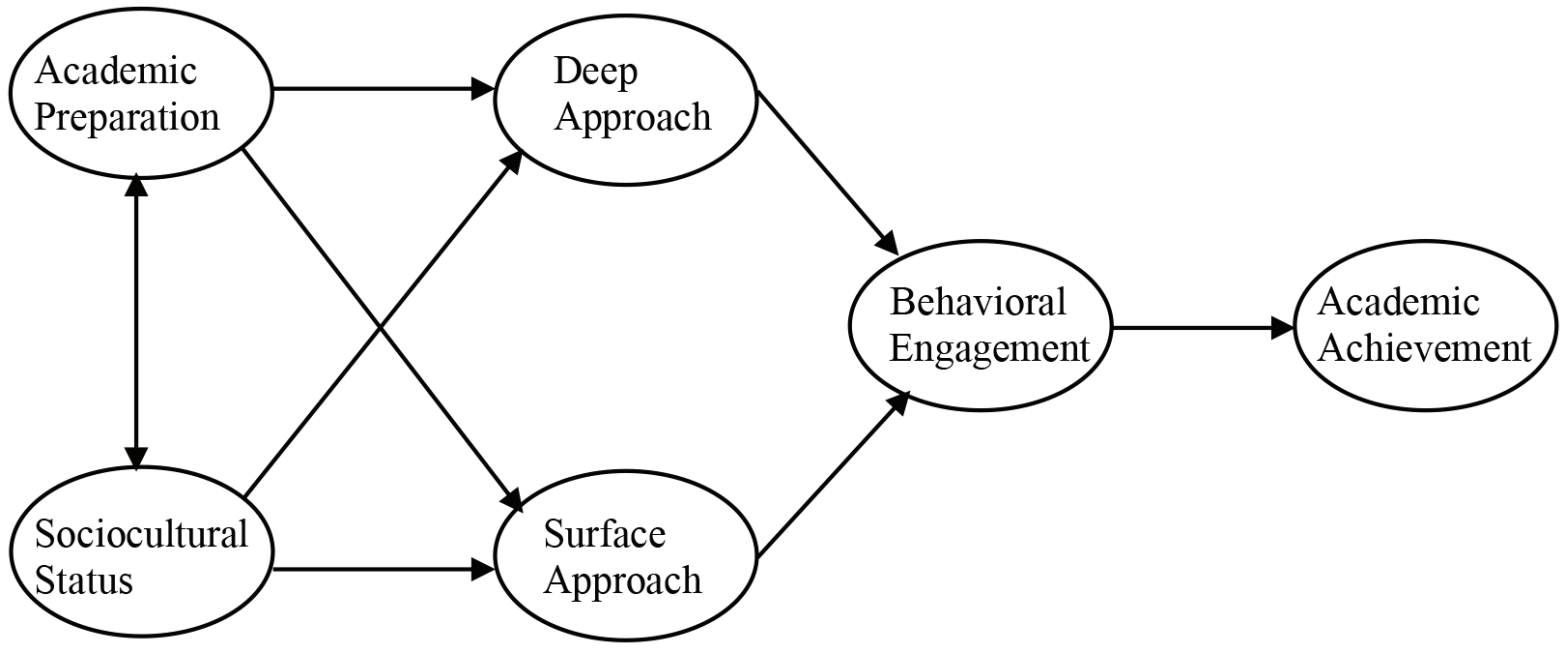
Hypothetical mediation model of student engagement in the first year of university.

The current study has two major goals as follows: analyze the mediation role of students’ engagement on the relationship between background variables (i.e., academic preparation and sociocultural status) and outcome variables (i.e., academic achievement) and analyze the invariance of the mediational model in the two time points. Based on the data available in the literature, the following hypotheses were set for the model both at the beginning and at the end of semester (Figure 1). Note that we used a one-tailed test for all because we were interested in analyzing results in a particular direction, accordingly to the literature:

H1: Students’ academic preparation is negatively correlated with the adoption of a surface approach and positively with the adoption of a deep approach to learning.

H2: Students’ sociocultural status is negatively associated with the adoption of a surface approach and positively with the adoption of a deep approach.

H3: Students’ behavioral engagement is negatively related to the adoption of a surface approach and positively to the adoption of a deep approach.

H4: Students’ behavioral engagement is positively associated with students’ academic achievement.

H5: No statistically significant differences are expected in the mediational model for the two time points (hypothesis of model invariance).

## Materials and Methods

### Participants

All students in their first year of a university in the north of Portugal were invited to participate in the study. One sample of 380 first-year students volunteered to participate at the beginning and at the end of the semester; 283 (73.9%) were female. Average age was 18.86 (DP = 2.13), ranging between 17 and 43 years old; 209 (55%) were first-generation students. Students were studying in different scientific areas: Biotechnology (27.3%), Health (27.1%), Psychology (10.5%), Law (23.2%), and Economics and Management (11.8%).

### Instruments and Measures

#### Predictor Variables: Academic Preparation and Sociocultural Status

– Academic preparation refers to knowledge and skills acquired during high school and refers to a latent variable in SEM with two indicators: language skills and high school grade point average (GPA). Language skills were assessed through an evaluation test of Portuguese Language skills, developed by experts, composed of a Reading Comprehension section (five items), a Grammar/Rewriting section (four items; e.g., *The students association deeply discussed the matter and finally proceeded to the vote.* R: *After…*), and a Logical Relations section (three items, e.g., *oneiric-dream*, *ludic-…*). Each correct answer was awarded 5 points, and each incorrect one received 0 points. At Time 1, the average score earned by the participants was 33.28 (DP = 10.18), with scores ranging from 10 to 60 points. At Time 2, the average was 32.73 (DP = 9.02), with scores ranging between 10 and 55 points. These tests were considered appropriate and valid to evaluate essential skills of the Portuguese language, through procedures of convergent and content validity (Trigo, 2012).
High school GPA was collected through academic services. In Portugal, high school scores in each discipline range from 0 to 20, with a passing grade of 10. High school GPA in Portugal corresponds to the mean of students’ high school grades across the three grade levels (60%) and the marks from national exams at the end of high school (40%). Minimum and maximum points were, respectively, 10.00 and 19.4. The average was 14.31 (DP = 1.82).
– Sociocultural status was also estimated in the model as a latent variable from two indicators: parental cultural capital and first-generation status. Parental cultural capital was adapted from De Graaf et al. (2000) and measured students’ participation in cultural activities (six items, Cronbach’s *α* of 0.82) and their reading habits (five items, Cronbach’s *α* of 0.76). Answers were rated on a 3-point Likert scale (1 = never, 2 = at least once a year, and 3 = more than once a year). The average score of participants’ answers was calculated in both scales, and the total of these two amounts was also calculated. The average of parental cultural capital was 3.67 (DP = 1.02). Minimum and maximum points were, respectively, 2 and 6 points.
First-generation status was assessed according to studies by Choi and Rhee (2014). A score of 1 meant students who reported that none of their parents had attended higher education institutions, and 2 signified students who reported that one or both of their parents had attended a higher education institution. There were 209 (55%) first-generation students.

#### Process Variables: Cognitive and Behavioral Engagement

– In this study, cognitive engagement was assessed through students’ approaches to learning, the surface approach being considered a sign of low cognitive engagement, and the deep approach exemplifying high cognitive engagement (Fredricks et al., 2004; Harris, 2011). Approaches to learning were assessed with the Study Processes Inventory for university students (Rosário et al., 2010), composed of 12 items, representative of two dimensions: surface approach and deep approach. Each dimension was composed of two subdimensions (motivation and strategy): the surface approach was composed of surface motivation and surface strategy, and the deep approach was composed of deep motivation and deep strategy. A recent study by Amieiro et al. (2018) has shown that this questionnaire has an acceptable reliability (parallel forms of Spearman-Brown): deep approach, 0.76; surface approach, 0.70. In the current study, the Cronbach’s *α* for surface approach scale was 0.70, and for deep approach, scale was 0.74. Respondents answered the questions through a 5-point Likert scale, from 1 (never) to 5 (always). For each one of the four subdimensions, the average of participants’ answers was calculated.
– Behavioral engagement was estimated as a latent variable from two indicators, namely study time and attendance at the lessons. These indicators were chosen because they are behaviors likely to represent students’ commitment with the academic work. The correlations found in Times 1 and 2 (Tables 1 and 2) between study time and class attendance support the option to include them in the same latent variable; moreover, we found high regression weights in the measurement model, which is likely to indicate that both are measuring the same construct: behavioral engagement.
Study time was measured by the average number of hours per week students spent studying one specific curricular unit (subject) selected by the researchers. Students rated the time spent studying during the week and weekend using a 5-point scale ranging from 1 (0 h) to 5 (4 h or more). The sum in both items was calculated. The average was 4.87 (DP = 1.55) and 5.51 (DP = 2.44) for Times 1 and 2, respectively. Scores ranged between 2 and 10 points for both times. Students’ class attendance was assessed summing the total of absences of participants in the selected curricular unit, until the moment of data collection. At Time 1, scores varied between 0 and 11, with an average of 0.98 absences (DP = 1.41). At Time 2, absences varied between 0 and 20, with an average of 2.17 (DP = 2.58). The answers were categorized and inverted, as 1 = 4 or more absences, 2 = 3 absences, 3 = 2 absences, 4 = 1 absence, and 5 = no absences.

**Table 1:** Correlation matrix and descriptive data (mean, standard deviation, skewness, and kurtosis) of observed variables included in the structural equation model in the first measurement time point (Time 1, beginning of the semester).

Measure	1	2	3	4	5	6	7	8	9	10	11
1	–										
2	0.64^**^	–									
3	0.20^**^	0.23^**^	–								
4	0.16^**^	0.15^**^	0.63^**^	–							
5	−0.27^**^	−0.28^**^	−0.08	−0.08	–						
6	−0.27^**^	−0.34^**^	0.08	−0.05	0.34^**^	–					
7	0.18^**^	0.17^**^	0.04	0.04	−0.08	−0.21^**^	–				
8	0.29^**^	0.40^**^	0.13^**^	0.03	−0.09	−0.28^**^	0.43^**^	–			
9	0.17^**^	0.31^**^	0.22^**^	0.11^*^	−0.18^**^	−0.24^**^	0.22^**^	0.27^**^	–		
10	0.24^**^	0.32^**^	0.18^**^	0.10	−0.24^**^	−0.23^**^	0.14^**^	0.31^**^	0.35^**^	–	
11	0.42^**^	0.53^**^	0.11^**^	0.02	−0.27^**^	−0.38^**^	0.14^**^	0.39^**^	0.36^**^	0.41^**^	–
*M*	33.28	14.31	3.67	1.45	2.96	2.56	3.78	3.30	4.87	4.09	2.98
SD	10.18	1.82	1.02	0.50	0.59	0.63	0.58	0.64	1.55	1.01	0.96
Skewness	0.13	0.27	0.01	0.21	−0.08	−0.03	−0.08	−0.04	0.14	−0.77	−0.21
Kurtosis	−0.35	−0.59	−0.65	−01.96	0.13	−0.56	−0.38	−0.28	0.22	−0.45	−0.12

*1, Language; 2, high school GPA; 3, parental cultural capital; 4, first-generation student; 5, surface motivation; 6, surface strategy; 7, deep motivation; 8, deep strategy; 9, study time; 10, class attendance; 11, academic achievement*.

* p < 0.05;

** *p < 0.01*.

**Table 2:** Correlation matrix and descriptive data (mean, standard deviation, skewness, and kurtosis) of observed variables included in the structural equation model in the second measurement time point (Time 2, end of the semester).

Measure	1	2	3	4	5	6	7	8	9	10	11
1	–										
2	0.37^**^	–									
3	0.12^*^	0.22^**^	–								
4	0.06	0.19^**^	0.51^**^	–							
5	−0.06	−0.12^*^	−0.20^**^	−0.09	–						
6	−0.10	−0.11^*^	−0.17^**^	−0.09	0.40^**^	–					
7	0.11^*^	0.13^**^	0.16^**^	0.09	−0.08	−0.07	–				
8	0.17^**^	0.18^**^	0.23^**^	0.15^**^	−0.12^*^	−0.12^*^	0.22^**^	–			
9	0.08	0.10^*^	0.13^*^	0.04	−0.14^**^	−0.13^*^	0.01	0.09	–		
10	0.07^*^	0.09	0.12^*^	0.02	−0.14^**^	−0.11^*^	0.13^*^	0.14^**^	0.23^**^	–	
11	0.28^**^	0.35^**^	0.13^*^	−0.02	−0.12^*^	−0.14^**^	0.21^**^	0.23^**^	0.21^**^	0.32^**^	–
*M*	32.73	14.37	3.80	1.48	2.82	2.56	3.69	3.43	5.51	3.27	3.01
SD	9.02	1.72	1.21	0.50	0.85	0.91	0.86	0.95	2.44	1.66	0.95
Skewness	0.08	0.36	−0.24	0.09	0.49	0.68	−01.16	−0.81	0.05	−0.27	−0.20
Kurtosis	−0.17	−0.13	−0.76	−01.99	0.38	0.46	1.83	0.56	−0.71	−01.61	−0.03

*1, Language skills; 2, high school GPA; 3, parental cultural capital; 4, first-generation student; 5, surface motivation; 6, surface strategy; 7, deep motivation; 8, deep strategy; 9, study time; 10, class attendance; 11, academic achievement*.

* p < 0.05;

** *p < 0.01*.

#### Outcome Variable: Academic Achievement

– Students’ academic achievement was evaluated based on the final grades of the selected curricular unit (subject) at the end of semester, through information given by academic services, after receiving participants’ consent. The average of grades was 10.91 (DP = 3.97) with scores ranging between 0 and 18. Owing to administrative reasons related to the protection of the privacy of students’ educational records, grade values were converted into the following five categories: 1 (from 0 to 4), 2 (from 5 to 9), 3 (from 10 to 13), 4 (from 14 to 16), and 5 (from 17 to 20).

### Procedures

To increase ecological validity and avoid too general operationalization of the variables (McKenzie et al., 2004; Plant et al., 2005), students’ cognitive and behavioral engagement and academic achievement were assessed using a specific curricular unit (subject) of the course as a reference. Curricular units of the different courses were selected according to two criteria: a core subject of the scientific area of the course and a subject with a high number of European Credit Transfer and Accumulation System (ECTS) credits (i.e., between 6 and 7.5 ECTS credits).

We carried out this study following the recommendations from the ethics committee at the University of Minho. All participants gave written informed consent to participate in the research in accordance with the Declaration of Helsinki. Participants were informed about the objectives of the investigation, and confidentiality and anonymity were assured (e.g., eliminating names and researchers’ personal notes that could link participants to their teachers or courses). In addition, participation in our study was voluntary, and researchers informed students about data usage. In both time points, data were collected in hard copy in the classroom, in time given by the teachers at the beginning or at the end of their classes, after authorizations of the administration of the university and participants’ informed consent.

### Data Analysis

The data were analyzed to verify that there were no values outside of the scale or missing values and to examine the linearity and normality of the measures. Five students were eliminated because they had a large amount of missing data or presented outlier values. No significant amount of missing data was found in any of the variables (in all cases <1.1%). The missing values were treated through the multiple imputation procedure. Therefore, the final sample contained 380 students. The data were analyzed in two steps. First, the model was adjusted for the data of the two measurement time points. Second, multigroup analysis was carried out to test the hypothesis of invariance.

The structural equation model was analyzed in two stages using the AMOS.22 program (Arbuckle, 2013). A series set of statistical and fit indexes were used to analyze the SEM model. Besides the chi-square (*χ*^2^) and its probability associated (*p*), the information given by Goodness-of-Fit Index (GFI) and the adjusted Goodness-of-Fit Index (AGFI), introduced by Jöreskog and Sörbom (1984); the Comparative Fit index (CFI; Bentler, 1990); the Tucker-Lewis Index (TLI), recommended by Hu and Bentler (1999); and the root mean square error of approximation (RMSEA), including confidence intervals (Browne and Cudeck, 1993) were used. The latter is recommended by MacCallum and Austin (2000) in the literature review about SEM applications. The effect size of the regression coefficients was calculated using Cohen’s (1988) *d* statistic.

## Results

### Initial Data Screening

Tables 1 and 2 present the matrices of correlations between the variables included in the SEM—Times 1 and 2—as well as the descriptive data, showing the totality of variables presented ideal asymmetry and kurtosis values. The majority of relationships between the variables were statistically significant at *p* < 0.001 (82% at the beginning of the semester and 48% at the end of the semester).

### Evaluation of the Mediation Model of Student Engagement

The global fit indices of the hypothesized mediation model of student engagement were good in both time points, Time 1 (*χ*^2^ = 73,575; gl = 37; *p* < 0.05; *χ*^2^/gl = 1,989; GFI = 0.967; AGFI = 0.940; CFI = 0.964; TLI = 0.947; RMSEA = 0.051; LO 90 = 0.034; and HI 90 = 0.068) and Time 2 (*χ*^2^ = 52,056; gl = 37; *p* < 0.05; *χ*^2^/gl = 1,407; GFI = 0.976; AGFI = 0.957; CFI = 0.968; RMSEA = 0.033; LO 90 = 0.010; and HI 90 = 0.052).

**Figure 2:**
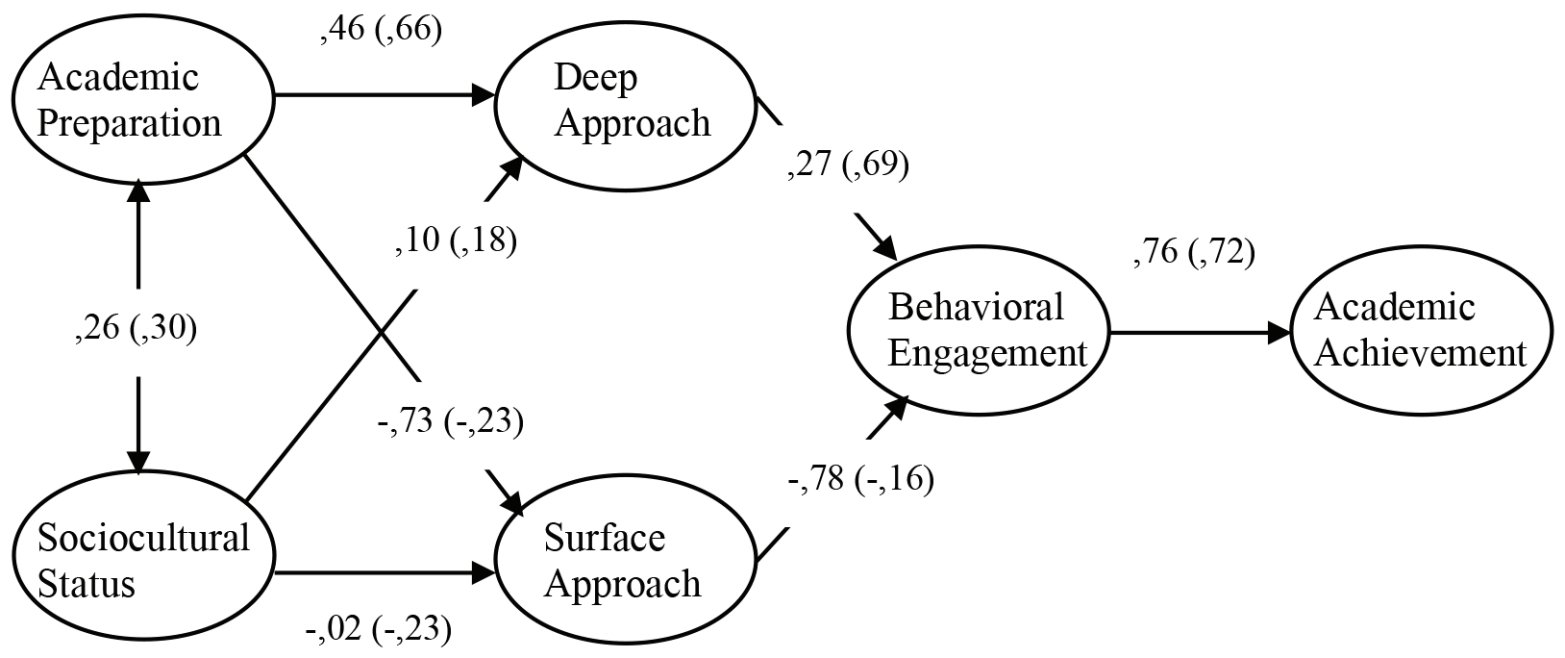
Hypothetical mediation model of student engagement in the first year of university. Standardized direct effects at the beginning (out of parentheses) and at the end of semester (inside the parentheses). Note: All parameters were statistically significant at *p* < 0.001, except sociocultural status and academic preparation on surface approach (at the end of semester), which were significant at *p* < 0.05. Sociocultural status on surface approach at the beginning of semester, sociocultural status on deep approach (at the two time points), and surface approach on behavioral engagement (at the end of semester) were not significant.

Overall, findings indicate the model fit in both time points; moreover, the detailed inspection to the modification indexes and residuals analysis did not suggest the need to include any significant relationships in the model. For this reason, we assumed that this as our final model. Table 3 presents the standardized estimated values for the structural equations referring to Times 1 and 2. Figure 2 summarizes the most relevant results for both time points.

**Table 3:** Standardized direct effects at the beginning (Time 1) and at the end (Time 2) of semester of the first year.

	Coefficients		Standardized errors		Critical ratio		Sign.	
	T1	T2	T1	T2	T1	T2	T1	T2
Academic preparation → surface approach	−0.733	−0.225	0.004	0.011	−6.732	−2.240	0.000	0.025
Academic preparation → deep approach	0.456	0.664	0.004	0.011	4.099	3.914	0.000	0.000
Sociocultural status → surface approach	−0.019	−0.225	0.016	0.045	−0.332	−2.324	0.740	0.020
Sociocultural status → deep approach	0.10	0.175	0.013	0.027	0.196	1.779	0.844	0.075
Surface approach → behavioral engagement	−0.782	−0.156	0.404	0.144	−5.463	−1.596	0.000	0.111
Deep approach → behavioral engagement	0.268	0.687	0.212	0.512	3.791	3.315	0.000	0.000
Behavioral engagement → academic achievement	0.760	0.719	0.113	0.190	8.281	4.468	0.000	0.000
Academic preparation ↔ sociocultural status	0.261	0.297	0.444	0.461	4.322	3.677	0.000	0.000

*T1, measurement at the beginning of semester; T2, measurement at the end of semester*.

Data in Figure 2 indicate that not all the hypotheses were supported. Specifically, the results support that students’ engagement completely mediated the relationship between background variables (i.e., academic preparation and sociocultural status) and outcome variables (i.e., academic achievement). Student engagement mediated the effect of academic preparation on academic achievement, both at the beginning of the semester (indirect effect: *b* = 0.529, *p* < 0.001) and at the end of the semester (indirect effect: *b* = 0.343, *p* < 0.001). Student engagement mediated the effect of sociocultural status on academic achievement through its relationship with academic preparation (*r* = 0.261 at the beginning of semester and *r* = 0.297 at the end of semester, see Table 3 and Figure 2).

The first hypothesis (H1) was supported for both measurement time points: at the beginning and the end of semester, the students’ academic preparation was negatively related to the adoption of a surface approach (the size of the effect was higher at the beginning of the semester, *d* = 0.736, than at the end of the semester, *d* = 0.231) and was positively related to the adoption of a deep approach (the size of the effect was moderate in both time points, *d* = 0.430 at the beginning and *d* = 0.410 at the end of semester). However, the second hypothesis (H2) was not supported in either of the two measurements: in general terms, the data indicate that sociocultural status was not related to student use of a particular approach to learning. The third hypothesis (H3) was supported in the first measurement time point, although not completely in the second measurement time point. As can be seen in Figure 2, despite the hypothesized relationships being in the expected direction (the more utilized a deep approach was, the higher the behavioral engagement would be, and the more a surface approach was utilized, the lower behavioral engagement was, and vice versa), the effect of the surface approach on behavioral engagement was not determined to be statistically significant. The size of the effect of the three statistically significant relationships was moderate (at the beginning of semester: surface and deep approach on behavioral engagement, *d* = 0.584 and *d* = 0.397, and at the end of semester: deep approach on behavioral engagement, *d* = 0.345). The fourth hypothesis (H4) was totally supported in both time points of measurement: behavioral engagement powerfully determined academic achievement at the beginning of semester (*d* = 0.938) and moderately determined student achievement at the end of the semester (*d* = 0.471), which allows for the conclusion to be made that the more the students engage in their academic tasks, the stronger their academic achievement will be.

Findings indicate that the mediational model explained 58% of the variability in academic achievement at the beginning of the semester and 52% at the end of the semester. On the other hand, student behavioral engagement was largely explained by the study approach, directly, and by academic preparation, indirectly (83% at the beginning of the semester), although to a lesser extent at the end of the semester (55%). Finally, the surface approach was more thoroughly explained by background variables (i.e., academic preparation and sociocultural status) at the beginning of the semester than at the end of it (55 and 13%, respectively), and the deep approach was more thoroughly explained at the end of semester (51%) than at the beginning (21%).

### Analysis of Temporal Invariance

The hypothesized mediational model has shown a good fit in both time points; however, some differences have been observed regarding the structural relationships in the model. Therefore, we proceeded to study these differences through multigroup analysis. Table 4 presents the results in relation to the invariance with respect to measurement weights, structural weights, structural covariances, structural residuals, and measurement residuals.

**Table 4:** Multigroup analysis.

Model	*χ*^2^(df)	*χ*^2^/df	*p*	AGFI	CFI	RMSEA (LO-HO)
Unconstrained	125.63 (74)	1.698	0.000	0.95	0.97	0.030 (0.021–0.039)
Measurement weights	132.60 (80)	1.658	0.000	0.95	0.97	0.029 (0.020–0.038)
Structural weights	179.08 (86)	2.082	0.000	0.94	0.94	0.038 (0.030–0.046)
Structural covariances	199.87 (89)	2.246	0.000	0.93	0.93	0.041 (0.033–0.048)
Structural residuals	204.81 (92)	2.226	0.000	0.93	0.92	0.040 (0.033–0.048)
Measurement residuals	723.67 (103)	7.026	0.000	0.83	0.58	0.089 (0.083–0.095)
Independence model	1600.50 (110)	14.55	0.000	0.58	0.00	0.134 (0.128–0.140)

In general terms, it can be said that the data in Table 4 do not supported the hypothesis of temporal invariance (H5). In particular, adopting a strategy of comparing nested models, data show that there were no statistically significant differences in measurement weights [Δ*χ*^2^_(6)_ = 6,971; *χ*^2^/gl = 1,161; *p* = 0.323; TLI = −0.003], but we found differences in the structural weights of both models [Δ*χ*^2^_(6)_ = 46,479; *χ*^2^/gl = 7,746; *p* < 0.001]. Given that nested models are studied, when statistically significant differences are found at this level, it is no longer needed to continue with the analysis through the following levels (structural covariances, structural residuals, and residual measurement). In conclusion, current data indicate that the relations between the latent variables in the model vary from the first to the second time point of measurement.

## Discussion

### Theoretical Implications

The current study focused on the role of student engagement as a mediating agent in the relationship between student background variables and student academic achievement. Student engagement was simultaneously analyzed in two dimensions (cognitive and behavioral), according to the calls in the literature (Fredricks et al., 2004). The model was tested at two different time points of the first semester because this is a period in which the analysis of the dynamics of engagement is very important (van der Meer et al., 2018).

In general, data supported the hypotheses that student engagement is a mediating variable of the relationship between students’ academic preparation and sociocultural status and students’ academic achievement. These data are relevant in that they help further explain why, in some investigations, the sociocultural status was not found as a determinant of academic performance (Anderton et al., 2016; Postiglione et al., 2017). Current data were collected at a private university where, in general, the families of the students may have a higher sociocultural status than that of the families of students at public universities. This should be taken in consideration while discussing findings, despite the fact that there is no conclusive data to demonstrate this. Moreover, results suggest that the effect of students’ academic preparation and sociocultural status on academic achievement may be mediated by variables other than the student’s engagement; for example, variables related to family conditions to facilitate a student’s commitment to learning or other design with the same variables (e.g., sociocultural status influencing academic achievement *via* academic preparation).

Consistent with the literature, students’ academic preparation was negatively associated with the reported use of a surface approach (Entwistle, 2009; Trigwell, 2010). However, the regression weight decreased considerably from the first to the second time point. Good academic preparation can be, initially, a protective factor even when a surface approach is reported; however, at the end of the semester, that protective factor faded. Academic preparation was positively related to the reported use of a deep approach, with regression weights similar at the beginning and at the end of the semester. These findings are also consistent with prior research, indicating that students with better academic achievement are more likely to adopt a deep approach (McKenzie et al., 2004; Diseth et al., 2010; Rosário et al., 2013a).

In the current study, sociocultural status had no association with the reported approaches to learning at the beginning of the semester. This finding was unexpected because the sociocultural status is likely to impact the way in which students approach their academic work (Phan et al., 2010). However, the literature about overcoming disadvantage in higher education can help explain these results, highlighting the role of aspirations for a better future and beliefs in the value of higher education as a means for social mobility (Visser and Gerharz, 2016; Khattab, 2018). The Portuguese high school context may also help explain these data. In fact, students in the last years of high school are focused on studying for the national exams because exam scores are important to college admission. Moreover, the majority of students in the current research, irrespective of their social status, reported to enroll in private lessons to help them prepare for the exams, which may have hindered the expected effect of cultural capital on students’ approaches to learning. At the end of the semester, sociocultural status showed a negative effect on the reported surface approach and a positive one on the reported deep approach, in spite of the regression weights being moderate (−0.28 and 0.27, respectively). In sum, at the beginning of semester, sociocultural status does not seem important; however, as the semester continues, sociocultural status seems to help students cope with the academic challenges of college (e.g., understand complex themes, study of extensive contents and the effort to reconcile multiple tasks).

Findings on the relationships between cognitive and behavioral engagement showed that the reported surface approach was negatively associated with students’ behavioral engagement at the beginning and at the end of semester. Thus, students focused on fulfilling the minimum requirements, which prioritize memorization of contents without their comprehension and integration, showed a low level of investment on academic tasks, being more absent from classes and studying a few hours per week. At the end of the semester, the association was also negative, but the regression weight was considerably lower. The temporal proximity with the final exams can contribute to a high behavioral engagement at the end of the semester, even by students that preferentially adopt a surface approach. On the other hand, when a deep approach was reported, it was found to be positively associated with behavioral engagement at the beginning and at the end of semester, although with different regression weights. This finding is consistent with the literature and reinforces the idea that adopting a deep approach implies active involvement of students. In fact, engaged students are likely to realize how important attending classes (Barattucci et al., 2017), interacting with teachers and classmates, and learning with questions, examples, and exercises can be for their learning. Current data suggest that students who report using a deep approach, even in the peak period of preparation of work and examinations, need more time to fully understand the learning contents because they relate concepts and integrate their knowledge into a meaningful whole.

Behavioral engagement was positively related to academic achievement, either at the beginning or at the end of semester, corroborating the results of other investigations (Kuh et al., 2008). Study time is intended to promote an active involvement in the learning process, through autonomous work that improves the quality of learning. In addition, interaction with teachers and colleagues is also a key piece of the learning process in college. While focusing on the curricular unit selected, and comparing it with the end of semester, the association between behavioral engagement and academic achievement was slightly higher at the beginning of semester and was considered a stronger predictor of achievement. Thus, some weeks after the first lessons, when students are still familiarizing themselves with a new institution, new teachers, and classmates, with different curricular units (each one with its contents, teaching and assessment methods), with a set of extracurricular activities, students’ interest in specific disciplines seemed to be positively correlated with impact on achievement at the end of the semester.

In summary, at the beginning of the semester, the variance in behavioral engagement was mostly explained by surface approach, while at the end of the semester, it was mostly explained by deep approach. Current data indicate primary and secondary paths for each of the time points. In the first time point, at the beginning of the semester, the primary path indicates that the lower the prior knowledge, the higher the use of surface approach, and the lower the behavioral engagement, while the secondary path indicates that the higher the prior knowledge, the higher the deep approach, and the higher the behavioral engagement. In the second time point, the paths are inverted: the primary path is through deep approach, and the secondary is through surface approach.

### Practical Implications

Results allow for the identification of important educational implications. Strong prior academic knowledge was related to students taking a deep approach to learning at the detriment of a surface approach; however, we learn from current data that the protective potential of strong prior academic knowledge seems to decline at the end of the first semester. Thus, students need to know and be able to recognize different approaches, identifying the factors that influence them to adopt a certain approach, and understand the advantages and disadvantages of their options. All considered, first-year students should be helped by faculty and by the educational services at college to understand and metacognitively control their approach to learning (Rosário et al., 2014a).

Data lead us to specifically worry about students who enter college with a low level of prior academic achievement and weak language skills. These students were more likely to adopt surface strategies, based on mechanical memorization of contents, which, as stated in the literature, are associated with lower levels of academic achievement (Herrmann et al., 2017). Concern over these students increases when we consider that this relationship seems to be very influential at the beginning of semester, so it seems to be relevant to identify the predominance of surface motivations and strategies as early as possible, to avoid academic failure and dropout in higher education.

In addition, considering the heterogenization of university student bodies, the use of student-centered models of teaching and learning and promoting student’s autonomy must be conciliated with the availability of support given by the institutions. In a world pledging for inclusion, universities are expected to be creative in responding to students’ learning needs, especially first-year students who are at most risk of failing. The following are examples of educational services that universities could consider offer to students: provide students with specific courses or programs that have a remedial effect on the difficulties detected (Cerezo et al., 2010; Rosário et al., 2013b), reinforce the mechanisms of supervising students (e.g., mentoring programs), create moments for students to increase knowledge about their own learning processes (e.g., catch-up content opportunities), and promote teachers’ awareness of how their curricular units’ characteristics and functioning can be promoting different ways of approaching learning tasks by students.

As we learn from the current data, socially disadvantaged students presented a higher risk of adopting learning methods that may lead to a low level of cognitive engagement. As the semester progresses and the demands increase, these first-year students are more likely to adopt extrinsic motivations, completing a minimum amount of required tasks, and using strategies centered on mechanical memorization of contents. According to James et al. (2010), some subgroups of students should be monitored closely, as the literature shows that the experience students have in their first year varies significantly depending on students’ backgrounds. Thus, universities should consider improving their mechanisms of collecting information to allow for early identification, support, and monitoring of students at risk of dropping out (Merchán et al., 2019), showing high level of disengagement and low academic achievement.

The possibility of analyzing the same model, with the same variables, at the beginning and at the end of the first semester, furthers our understanding of the role played by the distinct factors on students’ academic achievement. In fact, this role is not stable as the semester progresses, and this knowledge adds to the existing literature, but additionally to the faculty and educational services of the university. Current findings may inform the academic activities delivered by the universities to first-year students.

Finally, the current study points out the responsive nature of cognitive and behavioral engagement, assuming a systemic view in which different factors are closely interrelated. The analysis of both SEM also allows us to understand that academic achievement at the end of the semester is closely related to what happens at the beginning of the semester (e.g., approach to learning, study time).

Thus, promoting students’ engagement at the beginning of the semester should be considered a priority, as the first part of the first semester is a critical period for the students and for their integration to the university. For example, faculty could consider acknowledging students’ prior achievement and designing their teaching and learning activities to better match their learning needs (e.g., catch-up weeks, intensive leveling courses on language or math). In sum, it is important to carry out interventions specifically designed for first-year students (Rosário et al., 2014a; van der Meer et al., 2018) and to consider them a priority.

## Limitations and Directions for Future Research

In spite of the good fitting model at the beginning and at the end of semester, a significant amount of the variance has not been explained. For example, due to administrative reasons related to the protection of the privacy of students’ educational records, the variable academic achievement was converted in a five-item scale. This decision reduced the variability of data and may have contributed to current results. Moreover, and considering that our focus was on the student variables likely to explain academic achievement, future research could consider including variables focused on the teacher or on teaching context (e.g., approaches to teaching, value of teaching for faculty career; types of assessment). The inclusion of variables of a distinct nature focused on the teachers’ roles or on the teaching process could help increase the variance explained by the endogenous variables, thus contributing to a better understanding of the complex learning processes occurring during the first semester in college.

More investigation is needed to further understand the complex phenomena of approaching learning in the first semester in college, for example, examining the role played by teachers’ approaches to teaching (Rosário et al., 2013b, 2014b), the characteristics of the curricular units, the support and feedback delivered by faculty in class, and the teaching and learning microprocesses that occur in the classroom (e.g., number and type of questions asked in class by faculty and students) (Karagiannopoulou and Milienos, 2015; Jones, 2018; Lu and Wu, 2018; Ribeiro et al., 2019). Findings would shed light on the real class dynamics between students’ engagement and teachers’ styles, teaching practices, and behaviors (Reeve and Tseng, 2011). Another limitation concerns the fact that the study only included measures of cognitive and behavioral engagement. Future studies could consider including measures of emotional engagement (using recently published validated instruments such as University Student Engagement Inventory from Maroco et al., 2016), as well as other factors that contribute to student engagement. Moreover, the use of self-report measures does not allow for the capture of the processual nature of the constructs analyzed. Future studies could consider combining self-report measures with event measures in real contexts (Perry, 2002), investigating teaching, and learning natural context (e.g., observing students’ academic behavior in distinct learning situations).

The cross-sectional design of the current study impedes the assumption of causal relations, even using SEM. Future investigations could extend data collection for more than the first semester, to understand if the model fits in the second semester of the first year, and test the invariance of models to learn if the regression coefficients are significantly different from those observed in the first semester. Preference could be given to designs using multilevel analysis that work to determine the variance of academic achievement explained by variables of different levels simultaneously.

## Data Availability Statement

The datasets for this article are not publicly available due to stipulations laid out in the informed consent forms. Requests to access the datasets should be directed to Luisa Ribeiro, the corresponding author [lmribeiro@porto.ucp.pt].

## Ethics Statement

This study was reviewed and approved by the ethics committee of the University of Minho. All research participants provided written informed consent in accordance with the Declaration of Helsinki.

## Author Contributions

LR and PR contributed to the conception and the design of the work. LR and PR were responsible for data collection, analysis, and interpretation of data. LR wrote the manuscript with valuable inputs from the remaining authors. JN made important contributions to data analysis. MG and SF made important intellectual contributions to the paper. All authors agreed on all aspects of the work and approved the version to be published.

### Conflict of Interest

The authors declare that the research was conducted in the absence of any commercial or financial relationships that could be construed as a potential conflict of interest.

The reviewer LA declared a shared affiliation, with no collaboration, with one of the authors, PR, to the handling editor at time of review.
